# Astrocyte Role in Temporal Lobe Epilepsy and Development of Mossy Fiber Sprouting

**DOI:** 10.3389/fncel.2021.725693

**Published:** 2021-09-30

**Authors:** Carolyn Twible, Rober Abdo, Qi Zhang

**Affiliations:** ^1^Department of Pathology and Lab Medicine, Western University, London, ON, Canada; ^2^Department of Anatomy and Cell Biology, Western University, London, ON, Canada; ^3^Department of Pathology and Lab Medicine, London Health Sciences Centre, University Hospital, London, ON, Canada

**Keywords:** astrocyte, epilepsy, hippocampus, temporal lobe, pathology, mossy fiber

## Abstract

Epilepsy affects approximately 50 million people worldwide, with 60% of adult epilepsies presenting an onset of focal origin. The most common focal epilepsy is temporal lobe epilepsy (TLE). The role of astrocytes in the presentation and development of TLE has been increasingly studied and discussed within the literature. The most common histopathological diagnosis of TLE is hippocampal sclerosis. Hippocampal sclerosis is characterized by neuronal cell loss within the Cornu ammonis and reactive astrogliosis. In some cases, mossy fiber sprouting may be observed. Mossy fiber sprouting has been controversial in its contribution to epileptogenesis in TLE patients, and the mechanisms surrounding the phenomenon have yet to be elucidated. Several studies have reported that mossy fiber sprouting has an almost certain co-existence with reactive astrogliosis within the hippocampus under epileptic conditions. Astrocytes are known to play an important role in the survival and axonal outgrowth of central and peripheral nervous system neurons, pointing to a potential role of astrocytes in TLE and associated cellular alterations. Herein, we review the recent developments surrounding the role of astrocytes in the pathogenic process of TLE and mossy fiber sprouting, with a focus on proposed signaling pathways and cellular mechanisms, histological observations, and clinical correlations in human patients.

## Introduction

The definition of temporal lobe epilepsy (TLE) has not experienced significant revision since it was defined in 1985 by the International League Against Epilepsy (ILAE) as a condition characterized by recurrent, unprovoked seizures originating from the medial or lateral temporal lobe (ILAE, 1985). Focal-onset epilepsy, previously referred to as partial-onset, makes up approximately 60% of adult epilepsies, with TLE being the most common form of focal-onset epilepsy to be referred for surgical intervention due to being refractory to antiepileptic drugs (AEDs; Tellez-Zenteno and Hernandez-Ronquillo, 2011). TLE is further divided into mesial TLE (mTLE) and lateral TLE also known as neocortical TLE (nTLE), dependent on the structure in which epileptogenesis occurs (Tellez-Zenteno and Hernandez-Ronquillo, 2011). The temporal lobe is considered the most epileptogenic region within the brain and mTLE represents approximately 40% of adult epilepsies (Ladino et al., 2014). TLE lesions are most often a result of hippocampal sclerosis (HS), cortical development malformations, benign tumors, vascular formations, and post-infectious or post-traumatic gliosis (Cendes, 2005). Risk factors for TLE consist of febrile seizures, CNS infections, head trauma, and perinatal injuries (Ladino et al., 2014). TLE can be sporadic or familial and typical presentation of a seizure episode begins with lack of responsiveness and staring, often with hand or mouth automatisms (Ladino et al., 2014). To diagnose TLE, a series of medical tests and imaging are performed. Clinical semiology, or seizure signs and symptoms, is often the first step in epilepsy diagnosis and seizure localization followed by electroencephalogram (EEG) and/or various imaging techniques including magnetic resonance imaging (MRI), positron emission tomography (PET), and ictal perfusion single photon emission computed tomography (SPECT) (Ladino et al., 2014). Further evaluation through language and memory tests including neuropsychological evaluations, Wada test, and functional MRI (fMRI) can help to further localize and lateralize seizure activity (Sheth, 2002; Ladino et al., 2014). In 60% of TLE patients, appropriate seizure control can be achieved with AED treatment, while the remaining 40% of TLE patients are considered to have drug resistant epilepsy (DRE) (Kwan and Sander, 2004). TLE patients who are refractory to AED treatment may be candidates for surgical or neuromodulation treatments (Ladino et al., 2014). mTLE is one of the most common forms of epilepsy referred for surgical treatment (Tellez-Zenteno and Hernandez-Ronquillo, 2011).

The most common finding upon histological examination of tissues from drug-resistant TLE patients is HS (Blumcke et al., 2012). HS is characterized by pyramidal cell loss within any of the 3 Cornu ammonis (CA) fields as well as hilus, which can also be referred to as CA4, and is categorized into HS Types 1–3 (Blumcke et al., 2013). This neuronal cell loss is often accompanied by astrogliosis, which is revealed by a network of intense glial fibrillary acidic protein (GFAP) positive immunostaining (Blumcke et al., 2013). A fourth HS type, termed no-HS, shows reactive gliosis only, with no pyramidal cell loss observed (Blumcke et al., 2013). Additionally, dentate gyrus malformations, including granule cell layer duplication or dispersion as well as mossy fiber sprouting may be observed in HS tissue samples (Schmeiser et al., 2017).

The term mossy fiber was coined by Cajal (1893, 1911) due to the fibers’ similarity in appearance to Cajal’s previously discovered cerebellar mossy fibers. These fibers are the non-myelinated axons of granule cells, and make up the second connection of the trisynaptic model (Blaabjerg and Zimmer, 2007). Mossy fibers possess multiple large presynaptic boutons, which surround the postsynaptic thorny excrescences protruding from the CA3 pyramidal cell apical dendrites (Evstratova and Toth, 2014). The phenomenon observed in HS termed mossy fiber sprouting is characterized by the granule cell axons projecting back into the molecular layer of the dentate gyrus (Cavarsan et al., 2018).

Traditionally, astrocytes have been described as neuron-supporting cells. It has become abundantly clear that astrocytes provide a much wider range of roles within the CNS, including the regulation of neuronal synaptogenesis (Allen et al., 2012; Tsai et al., 2012; Chung et al., 2013). Astrocytes provide a complex range of functions for both the healthy and injured CNS. In the following sections, we will briefly review the anatomy and histology of the human mesial temporal lobe (MTL) structures, with a focus on hippocampus. This will be followed by discussion of common pathological findings of TLE, the current research related to the astrocytes role in TLE, mossy fiber sprouting, and clinical correlations.

## Human Mesial Temporal Lobe Structures and Temporal Lobe Epilepsy

The MTL is comprised of several distinct structures including the hippocampal formation, amygdaloid complex, perirhinal cortex, and parahippocampal cortex (Suzuki and Amaral, 2003, 2004). The hippocampal formation can be further divided into the entorhinal cortex, dentate gyrus, hippocampus proper, and subiculum (Kensinger and Corkin, 2008). The most anterior structure within the MTL is the amygdala, with the hippocampus sitting posterior to the structure (Stefanacci et al., 1996; Kensinger and Corkin, 2008). The entorhinal cortex is located within the anterior and medial area of the temporal lobe and receives strong projections from other MTL structures: the perirhinal and parahippocampal cortices (Amaral et al., 1984; Kensinger and Corkin, 2008).

The MTL is integral to the transfer of short-term memory to long-term memory, and is predominantly involved in episodic and declarative memory in spatial navigation and experienced events (Engel, 2003; Kensinger and Corkin, 2008). Based on the known role of the MTL, it is not surprising that substantial damage to the MTLs can result in amnesia and an inability to create and preserve new memories (Squire, 2009).

### Hippocampus

**FIGURE 1:**
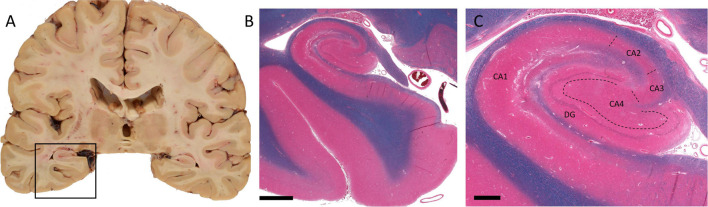
Macro- and microscopic images of human temporal lobe and hippocampus. **(A)** Coronal section of the cerebral hemispheres at level of lateral geniculate nuclei. The boxed area shows hippocampus and the parahippocampal gyrus. **(B)** Hematoxylin, eosin, and Luxol fast blue prepared histology section of the boxed area in **A**. **(C)** A higher magnification view of the hippocampal proper. Dashed lines represent boundaries of CA sectors. (DG, dentate gyrus; CA1–CA4, sectors of Cornu ammonis.) Scale bar in **B**: 3 mm, **C**: 800 μm. (Department of Pathology and Laboratory Medicine, London Health Sciences Centre, University Hospital, London, ON, Canada).

**FIGURE 2:**
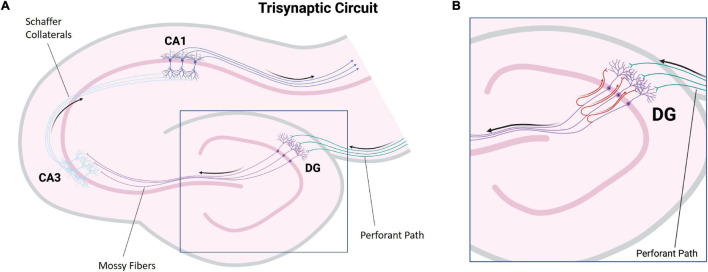
Illustration of major hippocampal connections. **(A)** A simplified diagram of the tri-synaptic circuit of the hippocampus. The entorhinal cortex provides synaptic input to the granule cells of the dentate gyrus *via* the perforant path (green). The granule cells project to CA3 pyramidal neurons *via* mossy fibers (purple). CA3 pyramidal neurons project to CA1 pyramidal neurons *via* Schaffer collaterals (light blue). CA1 pyramidal neuron axons carry hippocampal output (dark blue). **(B)** A diagram depicting mossy fiber sprouting. In hippocampal sclerosis associated mossy fiber sprouting, mossy fibers display aberrant growth and project back to the molecular layer of the dentate gyrus containing the apical dendrites of the granule cells (red). (DG, dentate gyrus: CA, Cornu ammonis) (Created with BioRender.com).

Lorente de No (1934) divided the hippocampus proper into subregions termed CA1, CA2, CA3, and CA4. The CA subregions contain pyramidal neurons as primary projection neurons, and each subregion is distinguishable by its pyramidal neuron morphology and morphometry (Figure 1; Knowles, 1992). Surrounding the CA4, also referred to as the dentate hilus, in a horseshoe shaped fashion is the dentate gyrus which acts as a gateway to the perforant pathway of the trisynaptic circuit (Figure 1; Knowles, 1992). Within the dentate gyrus, the typically compact granule cell layer is densely populated with mainly granule cells, the axons of which (termed mossy fibers) then project through the dentate hilus toward the dendrites of pyramidal cells in the CA3 subfield (Figure 2A; Blaabjerg and Zimmer, 2007). Recently, these mossy fiber collaterals have also been observed to make synaptic contact with dentate hilus and CA3 interneurons (Frotscher et al., 1994, 2006; Acsady et al., 1998; Henze et al., 2000). Within the CA3, mossy fibers innervate excitatory pyramidal cells, as well as inhibitory GABAergic cells (Acsady et al., 1998). Interestingly, a single mossy fiber contacts more GABAergic target cells than CA3 pyramidal cells, 30–50 and 10–20 respectively (Acsady et al., 1998). The higher level of contact with GABAergic cells indicates that there may be a role for granule cells in the regulation of CA3 pyramidal cell output (Szabadics and Soltesz, 2009). Aside from the granule cell layer, the mammalian dentate gyrus also contains the subgranular zone/polymorphic layer, located between CA4 and the granule cell layer, and the molecular layer, the “outer” layer of the dentate gyrus located toward the hippocampal sulcus (Cavarsan et al., 2018). In healthy brain tissue, the molecular layer is typically cell-free, containing apical dendrites from the adjacent granule cell layer which conduct signals from the entorhinal cortex and commissural projections through excitatory terminals (Cavarsan et al., 2018).

### Amygdala

The amygdala is located deep within the temporal lobe, in close proximity to the hippocampus. The amygdala is composed on several nuclei divided into the basolateral amygdala groups, cortical like groups, and centromedial groups (Sah et al., 2003). The amygdala has been shown to have a strong connection with the parahippocampal gyrus (including the hippocampus) in animal (McGaugh, 2000; McGaugh and Roozendaal, 2002) and human studies (Dolcos et al., 2004; Phelps, 2004; Smith et al., 2006) when processing emotional memory. Specifically, the basolateral amygdala groups have been shown to innervate primarily the CA1 sector of the hippocampus, with some projections identified in CA3 (Pikkarainen et al., 1999). The basolateral amygdala projections to CA1 have been suggested to be excitatory in nature (Felix-Ortiz et al., 2013; Yang et al., 2016).

### Temporal Lobe Epilepsy

**FIGURE 3:**
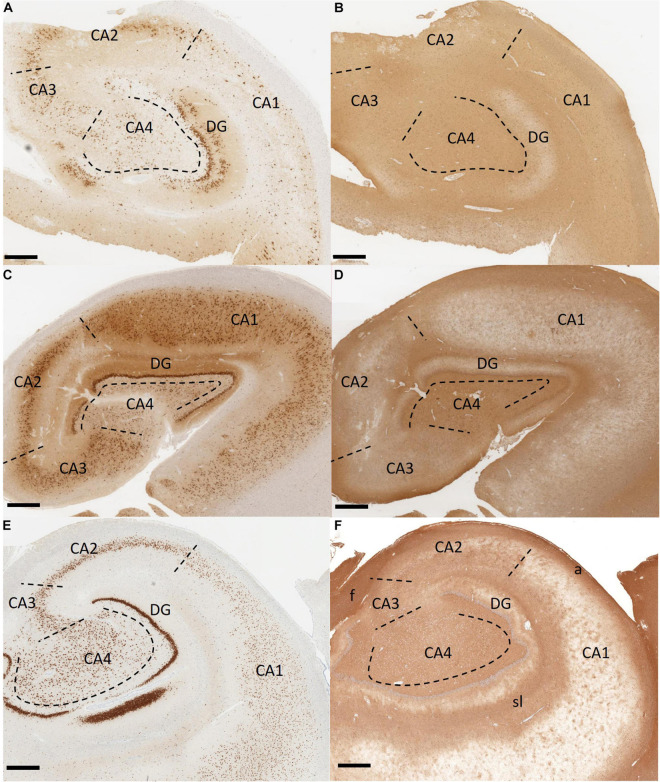
Immunohistochemistry stained histological sections of hippocampus tissue resected from patients with temporal lobe epilepsy. **(A,B)** An example of hippocampal sclerosis Type 1. There is significant pyramidal neuronal loss in CA1 and CA4. CA3 and dentate gyrus also show moderate to severe neuronal loss. Dentate granule cell dispersion and bilamination (“tram-tracking”) are also present **(A)**. There is diffuse reactive gliosis **(B)**. **(C,D)** Hippocampal sclerosis Type 4 (no-HS). There is no significant neuronal loss in the CA sectors **(C)**. Gliosis, more prominent in the CA4, is present **(D)**. **(E,F)** Non-epileptic hippocampus from an autopsy case. The CA sectors are well populated with neurons. Strong positive GFAP staining is localized in the white matter (alveus, fimbria, and striatum lacunosum). Non-specific staining is also noted in CA4 and CA3. Immunohistochemistry with antibodies to NeuN in **(A,C,E)** and GFAP in **(B,D,F)**. Scale bar 500 μm. (CA, Cornu ammonis; DG, dentate gyrus; a, alveus; f, fimbria; sl, striatum lacunosum. Department of Pathology and Laboratory Medicine, London Health Sciences Centre, University Hospital, London, ON, Canada).

Temporal lobe epilepsy is the most common form of focal-onset epilepsy to be referred for surgical intervention due to being refractory to AEDs, with the most frequent finding being HS (Tellez-Zenteno and Hernandez-Ronquillo, 2011; Blumcke et al., 2012). HS has been divided into four subtypes with varying degrees of sector-specific neuronal loss. This hallmark neuronal loss is always associated with severe astrogliosis, visualized by a GFAP-positive meshwork of processes (Blumcke et al., 2013). HS Type 1 is the most common form among HS surgical cases, making up approximately 60–80% in reported series (Bruton, 1988; Davies et al., 1996; Blumcke et al., 2002, 2007; de Lanerolle et al., 2003; Thom et al., 2010; Blumcke et al., 2013). In HS Type 1, pyramidal neuron loss is most severe in the CA1 sector with over 80% cell loss (Blumcke et al., 2012). Other sectors also show significant neuronal loss, specifically CA3 and CA4 with 30–90 and 40–90% neuronal loss, respectively (Blumcke et al., 2013). Outside of the CA sectors, neuronal loss can also affect the dentate gyrus with 50–60% granule cell loss in HS Type 1, which may be accompanied by varying types of granule cell pathology including granule cell dispersion and ectopic or bilayered granule cells (Figures 3A,B; Wieser, 2004; Blumcke et al., 2013). HS Type 2 is much less common, making up only 5–10% of surgical HS cases (Blumcke et al., 2013). In HS Type 2, pyramidal neuronal loss is predominantly found in CA1 affecting almost 80% of pyramidal cells while other CA sectors show mild neuronal loss (Blumcke et al., 2013). The dentate gyrus can also be affected in HS Type 2, however granule cell dispersion is the more common granule cell pathology while significant granule cell loss is uncommon (Blumcke et al., 2007). HS Type 3 is the rarest subtype, making up only 4–7.4% of surgical cases (Bruton, 1988; Blumcke et al., 2007; Thom et al., 2010). HS Type 3 is characterized by pyramidal cell loss predominantly in CA4 (approximately 50% cell loss) as well as the dentate gyrus (approximately 35% cell loss), while other CA sectors are only moderately affected with approximately 20–30% cell loss dependent on the CA sector (Blumcke et al., 2012). The fourth HS subtype, termed No-HS is arguably the most atypical due to the virtually intact pyramidal cell population in all of the CA sectors, with reactive gliosis only (Figures 3C,D; Blumcke et al., 2007). In non-epileptic hippocampus, the CA sectors are well populated with neurons and the positive GFAP immunolabelling is primarily localized in the white matter (Figures 3E,F). Approximately 20% of surgical TLE cases encounter this diagnosis, making it the second most common subtype of HS, yet relatively little is known surrounding the pathological mechanisms of this unique subtype. No-HS is of keen interest in terms of potential for glia-mediated seizure activity in TLE due to the gliosis-only histopathological diagnosis. HS surgical resection tissue may also present with sprouting of the granule cell axons, termed mossy fiber sprouting. Mossy fiber sprouting is thought to be triggered by the “injury *per se*,” which promotes neuronal activity and growth factor release, leading to the aberrant growth of the mossy fiber back into the molecular layer of the dentate gyrus (Figure 2B; Ikegaya, 1999; Binder et al., 2001; Koyama et al., 2004; Bekirov et al., 2008; Shibata et al., 2013; Song et al., 2015). The contribution of mossy fiber sprouting to epileptogenesis and chronic epilepsy has been debated within the literature, with some arguing for a compensatory role (Sloviter, 1992; Sloviter et al., 2006) and others a more epileptogenic role (Tauck and Nadler, 1985; Feng et al., 2003). Mossy fiber sprouting has also been argued to simply be an epiphenomenon of temporal lobe epilepsy (Gloor, 1997). Aside from HS, other findings upon histological examination of TLE surgical resection tissue include focal cortical dysplasia (FCD), vascular malformation, tumors and trauma (Al Sufiani and Ang, 2012; Blumcke et al., 2017).

**FIGURE 4:**
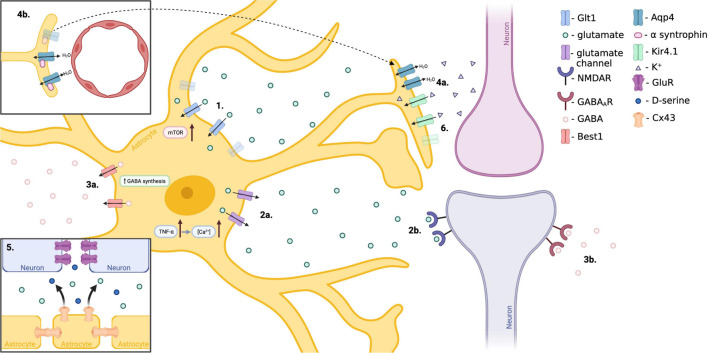
Proposed mechanisms of astrocyte involvement in TLE. (1.) Hyperactivation of mTOR within reactive astrocytes decreases Glt1 stability, resulting in reduced extracellular glutamate clearance and enhanced neuronal excitability. (2.) Increased expression of astrocytic TNFα results in excess release of glutamate from astrocytes in a Ca^2+^ dependent manner, affecting neuronal excitability. (3.) Reactive astrocytes over-produce and release inhibitory GABA through the Best1 channel as a compensatory mechanism to offset the excitatory shift of neurons in epilepsy. (4.) Reduced expression of alpha-syntrophin, responsible for anchoring adluminal Aqp4, results in mislocalization of adluminal Aqp4 to abluminal astrocytic endfeet. This mislocalization results in disrupted water homeostasis and is associated with delayed K^+^ clearance. (5.) Neuronal GluR expression is increased in TLE. Astrocytic Cx43 hemichannels release glutamate and D-serine resulting in pro-epileptic connexin activity. (6.) Loss of astrocytic Kir4.1 potassium channel results in disturbed K^+^ buffering and more positive neuron resting potential, enhancing neuronal excitability. (Created with BioRender.com).

## Astrocytic Functions in Normal and Diseased Central Nervous System

In the healthy CNS, an astrocyte network is present in virtually all regions and is organized in a neat, continuous, and non-overlapping manner (Sofroniew and Vinters, 2010). In gray matter, astrocyte-astrocyte contact is made through gap junctions (GJs) at the tips of astrocytic processes, and a similar form of contact is thought to exist in white matter (Bushong et al., 2002; Ogata and Kosaka, 2002; Nedergaard et al., 2003; Halassa et al., 2007b; Sofroniew and Vinters, 2010). Within the hippocampus, a single astrocyte’s processes are approximated to make contact with multiple neurons, enveloping over 100,000 synapses (Bushong et al., 2002; Ogata and Kosaka, 2002; Halassa et al., 2007b). Astrocytes exhibit sodium and potassium channels and display regulated increases in intracellular calcium concentrations (Cornell-Bell et al., 1990; Charles et al., 1991). Astrocytes’ regulated increases of intracellular calcium is suggested to have a functional role in communication between astrocytes and in astrocyte-neuron communication, including calcium elevations being triggered by transmitters released during neuronal activity and calcium elevations triggering release of transmitters from astrocytes into the extracellular space to elicit currents in neurons (Nedergaard et al., 2003; Volterra and Meldolesi, 2005; Halassa et al., 2007a; Shigetomi et al., 2008; Perea et al., 2009). Astrocytes have also been suggested to play a role in regulation of blood flow, synapse function, energy and metabolism, blood brain barrier (BBB), and fluid, ion, pH, and transmitter homeostasis (Sofroniew and Vinters, 2010). Early investigations suggest that structural, molecular, and possibly functional diversity in astrocytes exist, and longstanding evidence shows that the ratio of astrocytes to neurons approaches equality in organisms with more sophisticated neural tissues (i.e., worms 1:6 and humans 1.4:1), eluding to an increasingly important role for astrocytes (Nedergaard et al., 2003; Bachoo et al., 2004; Hewett, 2009).

In response to injury a diverse population of reactive astrocytes can be observed, varying between and within regions of the CNS (Giovannoni and Quintana, 2020). *In vivo*, Type A1 and A2 reactive astrocytes have been described, with A1 astrocytes displaying neurotoxic effects in response to injury while A2 astrocytes provide a neuroprotective role, however, it is suggested that additional subtypes of reactive astrocytes are present within the CNS (Giovannoni and Quintana, 2020). Local biochemical alterations promote astrogliosis surrounding a lesion following CNS injury (Sharma et al., 2015). Astrogliosis is a CNS defense response, which in its initial stages minimizes and repairs damage, but can subsequently result in harmful effects. Reactive astrogliosis, also termed astrocytosis, displays proliferation of astrocytes, characterized by hypertrophied processes and soma, and high expression of GFAP, nestin, and vimentin. The degree of astrogliosis can vary from mild to severe, with slight alterations in molecular profile and astrocytic hypertrophy or glial scar formation, respectively (Sharma et al., 2015). As the severity of astrogliosis increases, individual territories of astrocytes are lost with significant overlap of astrocyte domains, resulting in tissue distortion (Karimi-Abdolrezaee and Billakanti, 2012). Due to the many roles astrocytes play in the CNS, reactive astrocytes have the potential to impact a wide array neural activity through loss of essential functions and gain of detrimental effects. Reactive astrocytes can have detrimental effects such as aggravating inflammation, releasing possibly excitotoxic glutamate, compromising BBB function, and possibly contributing to seizure genesis (Brambilla et al., 2005; Jansen et al., 2005; Takano et al., 2005; Sanchez-Del-Rio et al., 2006; Argaw et al., 2009; Brambilla et al., 2009). However, reactive astrocytes have also been shown to repair the BBB, provide neural protection, and contain the spread of infection and inflammatory cells (Sofroniew and Vinters, 2010). Animal models suggest that disruption of reactive astrocyte function can attenuate glutamate uptake resulting in excitotoxic neurodegeneration or increased inflammation (Rothstein et al., 1996; Bush et al., 1999; Swanson et al., 2004; Voskuhl et al., 2009).

In regards to TLE, reactive astrogliosis is often prominent and has a virtually certain co-existence with HS, the most common histopathological finding upon examination of TLE surgical resection tissue (EngelJr., 1996). Within HS, astrocyte proliferation is most evident in regions of pyramidal neuron loss. Supporting the notion of astrocytic involvement in TLE is the use of AEDs that act on calcium signaling within astrocytes (Tian et al., 2005).

## Selected Cellular Mechanisms Pertinent to Temporal Lobe Epilepsy

Studies from TLE patients and related animal models have demonstrated alterations in expression and function of several cellular pathways expressed within astrocytes. These findings have implicated astrocyte dysfunction in neuronal hyperexcitation, neurotoxicity, and epileptogenesis or seizure spread (Steinhauser et al., 2012). Current knowledge and advances regarding mammalian target of rapamycin (mTOR) pathway, gliotransmission, astrocytic GABA, aquaporin 4 (Aqp4), GJs, and potassium regulation in relation to their potential contribution to TLE will be summarized.

### Mammalian Target of Rapamycin Pathway

The mTOR pathway is known to be a critical regulator of neuronal function, growth, and survival, and has been linked to numerous neurological diseases (Griffith and Wong, 2018). Within the CNS mTOR is expressed in brain endothelial cells, neurons, microglia, and astrocytes (Chong et al., 2012). Expression of mTOR within the cells of the CNS is kept at a relatively low level under normal physiological conditions, including within the astrocyte population (Codeluppi et al., 2009; Xu et al., 2010). The activity of the mTOR protein is influenced by various factors including: growth factors and insulin, mitogens, hormones, amino acids, and metabolism, and its expression can become highly upregulated when the CNS is experiencing injury (Chong et al., 2012).

The mTOR belongs to the family of phosphoinositide 3-kinase (PI3-K) related kinases (Latacz et al., 2015). This serine-threonine kinase is a 289 kDa multi-domain protein that is also referred to as FK506-binding protein 12-rapamycin complex-associated protein 1 (FRAP1) (Latacz et al., 2015). A single gene, termed FRAP1, is responsible for encoding mTOR in mammals (Brown et al., 1994; Chong et al., 2010). The function of this kinase protein relies on a catalytic subunit in mTORC1 and mTORC2 protein complexes (Chong et al., 2010; Benjamin et al., 2011). mTORC1 and mTORC2 are responsible for control of protein translation and control of cytoskeleton reorganization, respectively (Gingras et al., 1998; Sarbassov et al., 2004).

Increased mTOR activity has been suggested to play a role in the epileptogenesis of temporal lobe epilepsy, with inhibition of mTOR *via* rapamycin decreasing neuronal cell death, neurogenesis, mossy fiber sprouting, and the development of spontaneous epilepsy in a rat model (Zeng et al., 2009). Additionally, rapid and transient mTOR activation was observed in the hippocampus and neocortical tissue after status epilepticus was induced and was followed by a subsequent increase in mTOR during epileptogenesis and continued post-epilepsy onset within several rodent models (Buckmaster et al., 2009; Zeng et al., 2009; Huang et al., 2010). This suggests that epileptogenesis and chronic seizures are influenced by mTOR activity. Following these findings, Sha et al. (2012) found that expression of phosphorylated ribosomal protein S6 (pS6), an mTOR target, was increased within hippocampal granule cells and reactive astrocytes during the chronic phase of epileptogenesis using the intrahippocampal kainic acid model of TLE.

Interestingly, in human sclerotic hippocampi the mTOR signaling was found predominantly in reactive astrocytes, with only few cases presenting with mTOR induction in the small granule cell neurons (Sha et al., 2012; Sosunov et al., 2012). Using a transgenic mTOR knockout mouse model, deletion of mTOR from reactive astrocytes combatted the typical progressive increase in seizure frequency observed in epileptogenesis and reduced astrogliosis within the sclerotic hippocampus. However, no effect on aberrant mossy fiber reorganization was observed (Wang et al., 2017). The inhibition of mTOR resulted in the promotion of Glt1 stability *in vitro*, and in-turn, increased astrocyte removal of extracellular glutamate, suggesting a role for mTOR in regulating Glt1 stability in astrocytes through prevention of Glt1 degradation (Wang et al., 2017). Glt1 assists in maintaining extracellular glutamate levels below a neurotoxic threshold, and down-regulation of Glt1 in reactive astrocytes promotes a decrease in inhibitory synaptic transmission during epileptogenesis, suggesting enhanced excitability (Danbolt, 2001; Huang and Bergles, 2004; Regan et al., 2007; Petr et al., 2015). Therefore, it is possible that hyperactivation of mTOR within reactive astrocytes reduces extracellular glutamate clearance, potentially resulting in enhanced excitability and temporal lobe epilepsy progression.

The vascular growth factor (VEGF) receptor, VEGFR-3, has been observed as highly expressed within reactive astrocytes of patients with tuberous sclerosis and mTLE (Zhang et al., 2012; Sun et al., 2016; Castaneda-Cabral et al., 2019). This increased expression of VEGFR-3 within reactive astrocytes is mirrored in the pilocarpine-induced status epilepticus rodent models (Cho et al., 2019). Activation of VEGFR-3 has been recently reported to possibly induce PI3-K and serine-threonine kinase Akt pathways which are upstream activators of the mTOR pathway (Ruiz de Almodovar et al., 2009; Claesson-Welsh, 2016). VEGFR-3 immunoreactivity and mTOR activation demonstrate a positively correlated increase in pilocarpine-induced SE rodent model reactive astrocytes, and inhibition of both mTOR and VEGFR-3 resulted in attenuated Glt1 expression in hippocampal reactive astrocytes after SE (Jeong et al., 2021). This suggests VEGFR-3 upregulation-mediated mTOR induction within reactive astrocytes may be involved in GLT-1 expression in the hippocampus post-SE, leading to a reduction in neuronal hyperexcitability (Jeong et al., 2021).

The relationship between mTOR inhibition and Glt1 appears to be controversial, with some studies depicting a positive relationship in terms of extracellular glutamate clearance (Zeng et al., 2008; Wang et al., 2017), and others inferring a negative relationship (Jeong et al., 2021). This controversy could in part be due to the phase of epilepsy being investigated, as the negative relationship between mTOR inhibition and Glt1 expression was evident in a subacute phase of epilepsy (Jeong et al., 2021), while the positive relationship between mTOR inhibition and Glt1 stability was reported in a chronic phase of epilepsy (Wang et al., 2017). The mTOR pathway is now widely accepted to be involved in TLE, however, more research is needed to elucidate the relationship between mTOR activity within astrocytes and Glt1 expression.

Mammalian target of rapamycin hyperactivation has also been widely associated with mossy fiber sprouting in TLE. Rapamycin’s inhibitory effect on mTOR activity and subsequently mossy fiber sprouting has been demonstrated numerous times using various models of epilepsy (Zeng et al., 2009; Huang et al., 2010; Tang et al., 2012; van Vliet et al., 2012; Guo et al., 2013; Yamawaki et al., 2015; Hester et al., 2016). What has not been well established is the consequence of mossy fiber reorganization on seizure activity. Some studies have correlated mossy fiber reorganization with increased excitability (LaSarge et al., 2015), while others have suggested that aberrant growth of mossy fibers have no effect on seizure activity (Buckmaster and Lew, 2011; Heng et al., 2013).

### Gliotransmission

One of the major functions of an astrocyte is gliotransmission, or, the release of gliotransmitters (Riquelme et al., 2020). The neurotransmitters released by astrocytes regulate synaptic transmission and neuronal excitability through the functional unit formed by the astrocyte and neuron, termed the “tripartite synapsis” (Perea and Araque, 2007; Riquelme et al., 2020). Therefore, dysfunction in gliotransmission could have an effect on neuronal activity and a role in TLE *via* alterations in astrocytic neurotransmitters. Within the astrocyte-neuron functional unit, the astrocyte processes envelope the neuronal synapses, where gliotransmitters can be released dependent on Ca^2+^ concentrations (Riquelme et al., 2020). In non-pathologic CNS conditions, neuronal and glial release of adenosine triphosphate (ATP), glutamate, GABA and acetylcholine can subsequently cause an intracellular rise in Ca^2+^ within proximal astrocytes through activation of metabotropic receptors (Perea and Araque, 2007; Navarrete et al., 2012; Pascual et al., 2012). This Ca^2+^ rise within the astrocyte triggers the further release of gliotransmitters, including glutamate, ATP and D-serine, from the astrocyte processes within the tripartite synapsis (Perea and Araque, 2007; Mariotti et al., 2016). Heuser et al. (2018) found that within an epileptic mouse model, an increase in astrocytic Ca^2+^ waves correlated with an increase in glutamate release from astrocytes.

Within the tripartite synapse, glutamate can have both presynaptic and postsynaptic effects within excitatory and inhibitory neuronal networks (Fellin et al., 2004; Perea et al., 2016). Glutamate that is spontaneously released from astrocytes can trigger slow inward currents (SICs) postsynaptically, through NMDA receptors containing GluN2B on nearby neurons (Riquelme et al., 2020). In a rat model of chronic TLE, it was found that astrocytes within hippocampal slices presenting with astrogliosis displayed an increased occurrence of spontaneous slow Ca^2+^ transients (Alvarez-Ferradas et al., 2015). This Ca^2+^ transient pattern is suggestive of hyperexcitable astrocytes (Alvarez-Ferradas et al., 2015). A rise in SICs was also observed, indicating increased glutamate-mediated gliotransmission which up-regulates the basal probability of neurotransmitter release from CA3-CA1 synapses within the epileptic hippocampus (Alvarez-Ferradas et al., 2015). Therefore, in a Ca^2+^ dependent manner, astrogliosis-associated hyperexcitable astrocytes display an increased release of gliotransmitters that can affect neuronal excitability.

TNF-alpha (TNFα) is a proinflammatory cytokine that has been shown to regulate synaptic activity in the hippocampus through control of astrocyte glutamate release, and is also upregulated in epilepsy (de Bock et al., 1996; Avignone et al., 2008; Santello et al., 2011; Habbas et al., 2015; Patel et al., 2017). It has been observed that an increase in TNFα results in a rise in Ca^2+^ within the astrocyte (Nikolic et al., 2018). The effects of TNFα on Ca^2+^ levels are inhibited when a Ca^2+^ chelator is used to dialyze the astrocytes, indicating that TNFα triggers astrocyte glutamate release through a Ca^2+^ dependent mechanism (Nikolic et al., 2018). Previous work has implicated the purinergic receptor P2Y1 in the release of glutamate from astrocytes within CA1 (Shen et al., 2017). Blocking P2Y1 has been shown to inhibit Ca^2+^ astrocyte response triggered by TNFα, indicating a key role for P2Y1 in the activation of astrocytes by TNFα (Nikolic et al., 2018).

### Astrocytic GABA

The major cause of epilepsy has been suggested to be a shift in the neural network of the brain toward excitation, straying away from the normally balanced excitatory-inhibitory state (Sloviter, 1994; Maglóczky and Freund, 2005; Fritschy, 2008; Tóth et al., 2010; Huusko et al., 2015). This hypothesis however has previously struggled to explain the intermittent nature of epileptic seizures, that is, those experiencing epilepsy are not in a constant state of excitatory seizing (Muller et al., 2020). A possible explanation for the relatively rare seizure event is a compensatory mechanism that is capable of restoring the balance of excitation–inhibition within the brain for at least a portion of time (Walker and Kullmann, 2012; Pavlov and Walker, 2013; Staley, 2015). It has been hypothesized that reactive astrocytes, associated with many forms of epilepsy, aberrantly overproduce and release GABA which then activates high affinity, slowly desensitizing extrasynaptic GABA_A_ receptors (GABA_A_Rs) (Walker and Kullmann, 2012). In accordance with this hypothesis, recent work has implicated reactive astrocytes in the compensatory–inhibitory mechanism through the release of tonic GABA (Muller et al., 2020; Pandit et al., 2020). Tonic inhibition occurs through the activation of extrasynaptic GABA_A_R, in contrast to phasic inhibition occurring within the synapse causing a short, spatially restricted inhibition (Mody and Pearce, 2004; Semyanov et al., 2004; Farrant and Nusser, 2005; Lee and Maguire, 2014). Tonic inhibition is a more prolonged and continuous potent mode of inhibitory signaling, resulting in this form of inhibition being a candidate in mediating neuronal excitability (Mody and Pearce, 2004; Semyanov et al., 2004; Farrant and Nusser, 2005; Lee and Maguire, 2014). Astrocytic GABA has been reported as produced through monoamine oxidase-B and subsequently released through the Best1 channel to moderate tonic inhibition in multiple brain regions, including the hippocampus (Mody and Pearce, 2004; Semyanov et al., 2004; Farrant and Nusser, 2005; Lee and Maguire, 2014). This astrocytic tonic GABA effects brain function through regulation of the excitatory-inhibitory balance in both physiological and pathological conditions (Jo et al., 2014; Kim Y. et al., 2017; Woo et al., 2018). Interestingly, aberrant GABA has even been suggested as a useful molecular marker of reactive astrocytes (Chun and Lee, 2018). Using mouse models, it was found that Best1-mediated tonic inhibition inhibits CA1 neuronal firing and in turn suppresses seizure susceptibility (Pandit et al., 2020). Further, astrocyte-specific overexpression of Best1 within a Best1 KO mouse restores tonic inhibition and seizure susceptibility, strengthening the idea that astrocytic Best1 contributes to tonic inhibition by GABA release which suppresses seizure susceptibility (Pandit et al., 2020). Muller et al. showed a loss of hippocampal interneurons, thought to be responsible for a large portion of tonic inhibition, in a rodent model of TLE, yet interestingly found tonic inhibition levels to be the same between kainate-injected mice and control mice within the CA1 sector of the hippocampus (Glykys and Mody, 2007; Muller et al., 2020). However, within dentate granule cells, tonic currents were found to be higher in kainate-injected mice than controls, indicating preserved as well as increased GABA levels in the epileptic hippocampus despite GABAergic interneuron loss (Muller et al., 2020). In this study, astrocytes were found to display pronounced GABA accumulation and, similar to Pandit et al.’s (2020) finding, suggested GABA accumulation through synthesis rather than extracellular uptake (Muller et al., 2020). This data supports the notion that a compensatory mechanism exists to remediate the excitatory shift of the neural network in epilepsy, and that the occurrence of a seizure is possibly a periodic failing of this less stable compensatory mechanism (Pavlov and Walker, 2013; Muller et al., 2020). This possibly protective role of GABA released by reactive astrocytes presents a possible drug therapy target to explore.

### Aquaporin 4

Excessive extracellular potassium is generally accepted to enhance seizure susceptibility, and is a possible implication of Aqp4 in epilepsy due to its critical role in potassium clearance (Dudek and Rogawski, 2005). Aqp4, the most abundant aquaporin in the CNS, is a perisynaptic water channel predominantly expressed on astrocytic endfeet, anchored by the dystrophin complex (Neely et al., 2001; Dudek and Rogawski, 2005). The function of astrocytic Aqp4 is to transport water away from neuropil during periods of high neuronal activity, resulting in shrinkage of the extracellular space (Dudek and Rogawski, 2005). This water is fluxed toward the subarachnoidal space where the water is temporarily stored (Dudek and Rogawski, 2005). Neuronal firing also results in an increase in extracellular potassium, which under normal conditions, is cleared by astrocytes in a process coupled with the flux of water through Aqp4 (Nagelhus et al., 1999; Ostby et al., 2009; Haj-Yasein et al., 2012). Therefore, Aqp4 is thought to be integral in brain ion and water homeostasis during periods of high neural activity (Amiry-Moghaddam and Ottersen, 2003). Suboptimal potassium clearance by astrocytes leads to increased levels of potassium within the extracellular matrix (ECM), potentially contributing to seizure susceptibility. In the human sclerotic hippocampus, an increase in Aqp4 has been observed while expression of the anchoring protein dystrophin is decreased (Lee et al., 2004). This observation led to the hypothesis that the decrease in dystrophin results in a mislocalization of astrocytic Aqp4, resulting in disturbed water and ion homeostasis (Lee et al., 2004; Eid et al., 2005). The increase in Aqp4 levels could be due to TLE-associated gliosis, and therefore a result of an increased number of astrocytes within the epileptic brain. The hypothesized disturbed ability of Aqp4 to flux water away from the neuropil toward the subarachnoid space is supported by the increased edema observed in brain imaging of the sclerotic hippocampus (Lee et al., 2004). In latent-phase kainic-acid (KA) treated rats, Aqp4 density in astrocyte adluminal (facing capillaries) endfoot membranes was decreased, while the Aqp4 density in abluminal (facing neuropil) endfoot membranes was stable or slightly increased (Alvestad et al., 2013). Loss of perivascular Aqp4, *via* deletion of Aqp4 anchoring protein alpha-syntrophin, results in delayed extracellular potassium clearance, swelling of perivascular astrocyte endfeet, and enhanced seizure intensity (Amiry-Moghaddam et al., 2003). Alpha-syntrophin (a dystrophin associated anchoring protein) was observed to be reduced in the KA treated rats in the latent phase of epilepsy (Alvestad et al., 2013). Alpha-syntrophin deletion has been specifically observed to affect adluminal Aqp4 levels, while abluminal Aqp4 remains relatively stable (Amiry-Moghaddam and Ottersen, 2003). The association between adluminal Aqp4 and alpha-syntrophin likely explains the Aqp4 redistribution rather than loss. A decrease in Aqp4 has been observed during the latent phase of epilepsy, followed by a significant increase in Aqp4 protein levels 30 days pose SE (Hubbard et al., 2016). A previous study observed a similar reduction in Aqp4 immunoreactivity in the early phase of epilepsy (Lee et al., 2012). Another interesting observation is that Aqp4 expression was regulated by seizure activity in the contralateral hippocampus as well, where no sclerotic changes occurred (Hubbard et al., 2016). This indicates that regulation of Aqp4 by seizure activity does not appear to require cell death or sclerosis to be present in the tissue (Hubbard et al., 2016). Taken together, this evidence indicates that Aqp4 dysregulation may take place early in the epileptogenic process, disrupting ion and water homeostasis, and potentially contributing to epileptogenesis (Dudek and Rogawski, 2005; Lee et al., 2012; Alvestad et al., 2013; Hubbard et al., 2016). The dysregulation of Aqp4 carries over to the chronic phase of epilepsy, however, the pathological mechanisms in both phases of epilepsy remain to be further elucidated.

### Gap Junctions

Coordination in the neuronal network plays a crucial role in epileptic electrical activities, mainly through GJ channels that are the backbone for intercellular electrical coupling in the central nervous system (Hamidi et al., 2014). Astrocytes express many ion channels and transmitter receptors (Verkhratsky and Steinhäuser, 2000) and are functionally conjugated to other astrocytes and to oligodendrocytes by GJs (Kettenmann and Ransom, 1988; Butt and Ransom, 1989; Robinson et al., 1993; von Blankenfeld et al., 1993; Venance et al., 1995; Pastor et al., 1998). The intercellular permeability between astrocytes facilitates the diffusion of metabolites throughout the CNS tissues (Valiunas et al., 2005). The diameter of a GJ channel is large, allowing the diffusion of ions and molecules up to 1,000 Da. This permits the penetration of many molecules that function as second messengers, such as Ca^2+^, inositol trisphosphate (IP 3), cyclic adenosine monophosphate (cAMP), and important metabolites such as glucose, ATP, and their degraded products (Neijssen et al., 2005). The primary GJ proteins in astrocytes are connexin 43 (Cx43), which is the most abundant astrocytic connexin throughout the brain, including hippocampus (Gosejacob et al., 2011). Cx30, Cx26, Cx40, Cx45, Cx46, and Cx47 have also been shown to be expressed in these cells (Dermietzel et al., 1989; Yamamoto et al., 1990; Rash et al., 2001; Zahs et al., 2003; Altevogt and Paul, 2004). Neurons, particularly GABAergic interneurons, have GJs predominantly consisting of Cx36 in various brain regions (Rash et al., 2001). Connexins are glycoproteins that have four transmembrane domains, two extracellular loops (ELs), one cytoplasmic loop (CL), and a C- and N-terminal tail (CT and NT); six connexins oligomerize into a hemichannel (or connexon), and two hemichannels on opposing cell membranes form the intercellular channel, or GJ (Delvaeye et al., 2018). They are represented by 21 different isotypes in the human genome and 20 isotypes in the mouse genome (Willecke et al., 2002; Söhl and Willecke, 2003).

Gap junctions have a complex and controversial role in epilepsy and two possibilities have emerged. Studies of cerebral tissue from patients with epilepsy show upregulation of glial, but not neuronal connexins, strengthening the hypothesis that glial connexins are involved in seizures (Mylvaganam et al., 2010). It has been shown that increased Cx43 mRNA expression in temporal lobe neocortex from patients with refractory seizures and in neocortex from brain tumors that trigger seizures (Naus et al., 1991). Elevated Cx43 protein levels were identified in mesial-temporal lobe epilepsy (MTLE) patients (Fonseca et al., 2002; Collignon et al., 2006; Yao et al., 2009; Das et al., 2012). In contrast, decreased Cx43 mRNA was revealed in hippocampal tissue from patients with a complex partial seizure disorder (Elisevich et al., 1997). In KA-induced mouse model, there was a decrease in Cx30 expression within 12 and 24 h in the hippocampus while it is associated with an upsurge of Cx30 expression in cerebral cortex within 6 h of injection (Condorelli et al., 2002). When the junction blockers (octanol, carbenoxolone, and propionic acid) were administered in rat hippocampal slices, they reduce the duration of seizure that is induced by tetanic stimulation of Schaffer collaterals; nonetheless, ammonium chloride, which opens junctional coupling, elicits spontaneous secondary after-discharges (Jahromi et al., 2002). Nonetheless, Cx43 mostly has a role in pro-epileptic activity by releasing Glu and the NMDA receptor co-agonist D-serine (Abudara et al., 2018). When hemichannels open, they release multiple substances, including Glu, D-serine, and ATP (Xing et al., 2019). Glu receptor expression has been shown to express higher in TLE patients than in non-epileptic control (Mathern et al., 1997). Therefore, Glu and D-serine efflux cause significantly higher excitatory impulses in TLE.

Astrocytes also express pannexins which show significant homology with innexins, the proteins that form GJs in invertebrates (Panchin et al., 2000). Unlike connexins, pannexins are glycosylated (Penuela et al., 2007). Of the three mammalian pannexins, pannexin1 and pannexin2 are expressed in the CNS. Pannexin1 is unlikely to form functional channels but may form functional hemichannels (Locovei et al., 2006; Penuela et al., 2007). Some studies have demonstrated that Panx1 plays important role in regulating epilepsy. It has been documented that depletion of astrocytic Panx1 enhances while the absence of neuronal Panx1 constricts seizure manifestations in mice, suggesting different roles of astrocytic and neuronal Panx1 in epileptogenesis (Wang et al., 2018). Dossi and Blauwblomme (2018) found that the Panx1 channel promotes the origination and preservation of epilepsy through ATP signaling *via* purinergic 2 receptors. In addition, Panx1 expression was detected to be positively correlated with the seizure frequency in patients with FCD (Li et al., 2017). In comparison to Panx2, the expression of Panx1 protein is significantly increased in the temporal cortex of TLE patients than the normal group, indicating that the Panx1 channel may be involved in the pathogenesis of TLE (Jiang et al., 2013).

### K+ ion Regulation

During periods of neuronal activity, fluctuations in extracellular potassium (K+) concentration occur (Steinhauser et al., 2012). Increases in extracellular K+ concentration can result in a more positive resting potential and alterations in transmembrane ion channels, receptors, and transporters (Steinhauser et al., 2012). *In vivo*, extracellular K+ concentration shows substantial increase during neuronal hyperactivity, similar to that observed during epileptic activity (Steinhauser et al., 2012). This observation eludes to a role for increased extracellular K+ concentration in the pathogenesis of epilepsy. Astrocytes express a K+ channel, Kir4.1, that acts to influx K+ and has been shown to have a substantial role in glial K+ buffering (Kofuji et al., 2000; Djukic et al., 2007; Hibino et al., 2010). Kir4.1 knockout mouse models show deficits in size, weight, movement, and an epileptic phenotype (Kofuji et al., 2000; Djukic et al., 2007).

Kir4.1 and Aqp4 are both localized to the astroglial endfeet, indicating a spatial and potentially functional relationship between the transmembrane channels (Higashi et al., 2001). Mouse models with a decreased number of Aqp4 channels show impaired extracellular K+ clearance, and complete Aqp4 knockout show similar impairment and a prolonged seizure duration (Amiry-Moghaddam et al., 2003; Binder et al., 2006). A more recent study has shown functional independence of the Kir4.1 channel and Aqp4, with Aqp4 null mice showing no significant differences in membrane potential, Kir4.1 current, Kir4.1 protein expression, or Kir4.1 unitary conductance (Zhang and Verkman, 2008). The functional relationship between Kir4.1 and Aqp4 has yet to be fully elucidated.

Within TLE associated HS, studies have proposed that altered Kir channel expression has resulted in the observed K+ buffering impairment (Steinhauser et al., 2012). In human cases of HS, loss of astrocytic Kir4.1 immunoreactivity was observed and most significant surrounding vessels (Heuser et al., 2012). This loss in Kir4.1 immunoreactivity was restricted to gliotic areas of the hippocampus and associated with loss of alpha-syntrophin and dystrophin (Heuser et al., 2012). Patch-clamp recordings have also shown a reduction in Kir currents within the hippocampus of mTLE patients (Hinterkeuser et al., 2000). In contrast, some studies have shown an increased expression of Kir4.1 in human HS lesions (Aoki et al., 2019). Due to the known role of astrocytic Kir4.1 in extracellular K+ clearance, these changes likely result in disrupted K+ buffering and possibly contribute to seizures experienced by HS patients.

## Role of Astrocyte in Mossy Fiber Sprouting

Recently, astrocytes have been revealed to play a role in regulating synaptic connections in the CNS (Kim S. et al., 2017). Astrocytes contribute to synapse formation, elimination, and maturation, in the CNS developmental period as well as under pathologic conditions (Kim et al., 2016; Stogsdill and Eroglu, 2016). Specifically, thrombospondin (TSP)-1, an ECM protein derived from astrocytes, has been found to promote excitatory synapse formation during nervous system development and is subsequently severely downregulated in the mature brain (Christopherson et al., 2005; Eroglu et al., 2009). An exception to the observed downregulation of astroglial-derived TSP-1 in the adult brain is within regions of neurogenesis, including the subgranular zone of the dentate gyrus (Jones and Bouvier, 2014). Upregulation of TSP-1 reappears in the mature brain upon CNS pathologic insult, as astrocytes revert back toward immature signaling (Risher and Eroglu, 2012; Kim S. et al., 2017). TSP-1 is upregulated in astrocytes following seizure activity, as observed in a rat model of electroconvulsive seizure, where the total number of synapses in the mature rat hippocampus also increased (Chen et al., 2009; Okada-Tsuchioka et al., 2014). Studies have shown that the synapses formed by aberrant mossy fiber sprouting back toward the dendritic spines of other granule cells are excitatory (Represa et al., 1993; Hendricks et al., 2017; Cavarsan et al., 2018). Although these adult-generated synapses have since been deemed as likely non-functional (Hendricks et al., 2017), and more so an epiphenomenon of epilepsy related HS (Cavarsan et al., 2018), seizure induced upregulation of this ECM protein and the observed excitatory synapse formation by aberrant mossy fibers post-seizure activity perhaps presents a possible role for TSP-1 in the largely unknown cellular mechanism of mossy fiber sprouting. Another intriguing protein is tenascin (TN)-C, involved in cell migration, proliferation, axonal guidance and synaptic plasticity, and expressed by subpopulations of astrocytes (Jones and Bouvier, 2014). A similar link between TN-C expression and TLE has been previously described, and associated with reactive gliosis, synaptic reorganization, and axonal sprouting (Scheffler et al., 1998). TN-C follows a similar expression trajectory as TSP-1, being highly expressed during CNS development and subsequently downregulated in the adult brain, to again have expression reappear upon CNS injury (Jones and Bouvier, 2014). These observations present a possible implication of reactive astrocytes in the pathogenesis of HS-associated mossy fiber sprouting.

## Clinical Correlations

The kainic acid and pilocarpine animal models are commonly used in research due to similarities the models share with human TLE. These models use systemic administration of chemoconvulsants that result in an initial precipitating injury followed by a latent period and development of recurrent seizures with temporal origin, imitating the progression of human TLE (Levesque et al., 2016). These predominating animals models reproduce the clustering of seizures and histopathological findings, including loss of pyramidal neurons within the CA sectors of the hippocampus, granule cell layer dispersion, and mossy fiber sprouting in the dentate gyrus, observed in TLE patients (Mello et al., 1993; Cavalheiro, 1995; Arida et al., 1999; Grabenstatter et al., 2005; Haut, 2006; Goffin et al., 2007; Sharma et al., 2007; Williams et al., 2009; Bortel et al., 2010; Levesque et al., 2011; Drexel et al., 2012; Levesque et al., 2016). Of note, in some animal model studies of TLE neuronal degeneration occurs primarily in CA3, while in human disease CA1 is more commonly affected (Levesque et al., 2016). The kanic acid and pilocarpine models also reproduce the overall result of pharmacological treatment observed in human TLE with AEDs having a similar anti-seizure effect, recurrence of seizures upon AED cessation, as well as AED resistance in some animals (Levesque et al., 2016). Despite the overall similarities between the kanic acid and pilocarpine animal models and human TLE, limitations of these models do exist. The response to kainic acid and pilocarpine injection differs with injection placement, gender, age, and strain of animals used (Bragin et al., 1999, 2005; Curia et al., 2008; Zhang et al., 2008; Levesque and Avoli, 2013). This inter-laboratory difference may create difficulty interpreting overall findings within animal models of TLE. Prevalence of seizure type is also altered in animal models of TLE. Human TLE patients display seizure onset patterns that are either a LVF (diffuse onset zone) or HYP (focal onset zone) pattern, while animal models alternate between seizure onset patterns (Velasco et al., 2000; Bragin et al., 2005; Levesque et al., 2012). Pilocarpine model studies have also suggested that in addition to distinct onset zones, the mechanisms of generation between the two onset patterns potentially differ, creating additional complexity in applying animal model findings to human disease (Levesque et al., 2016).

Given that astrocytes may be implicated in the epileptogenesis of TLE, demonstrated by the previously discussed cellular mechanisms, examining the relationship between gliosis and clinical information is quite intriguing. Few studies have been published investigating the relationships between extent of gliosis in TLE and clinical information, such as post-surgical outcome. In the limited studies that have been conducted, contradictory results have been observed. GFAP expression has been positively correlated to seizure frequency in a small study of HS (*n* = 12) (Cohen-Gadol et al., 2004), and conversely, no significant correlation between degree of astrogliosis and clinical information was found in another similar study (Blanc et al., 2011). The relationship between degree of gliosis and post-surgical outcome of TLE patients has received even less attention, as prior to a study done by Johnson et al. (2016), only one study had examined the relationship, yet found no correlations between the glial density of mTLE patients (*n* = 62) and seizure freedom (Spencer et al., 1999). However, in the study conducted by Johnson et al. (2016), HS patients that had a poor post-surgical outcome (Engel III or IV evaluated at 1-year post-operation) demonstrated a significantly higher number of astrocytes in CA3 and lower numbers of astrocytes in the lower cortex. Based on these findings, it was hypothesized that the observed increase in CA3 astrocyte reactivity potentially reflects an increase in reactivity of hippocampal projection pathways (trisynaptic circuit) (Johnson et al., 2016). Johnson et al. also re-demonstrated the previous findings of GFAP correlation with seizure frequency (Cohen-Gadol et al., 2004; Johnson et al., 2016). More specifically, reactive astrogliosis (defined by increased levels of GFAP-IR density) in CA1 and increased astrocyte number in CA2 were significantly associated with a high seizure frequency (Johnson et al., 2016).

Among histopathologically examined TLE cases, approximately 20% do not display any neuronal loss but do display varying levels of reactive gliosis, the group termed “no-HS” (Blumcke et al., 2002, 2007; Thom et al., 2005). This group of TLE cases is particularly interesting in the context of astrocyte contribution to epileptogenesis, considering gliosis is the only notable observation with standard diagnostic practices. As for post-surgical seizure freedom within no-HS patients, results are seemingly variable. Studies investigating this relationship have observed seizure freedom rates between 20 and 58.6% (Bruton, 1988; Davies et al., 1996; de Lanerolle et al., 2003; Blumcke et al., 2007; Thom et al., 2010). The studies with the highest no-HS sample sizes, 38 and 34, also had the most variable seizure freedom observations, 20 and 58.6%, respectively (Bruton, 1988; Blumcke et al., 2007). Interestingly, a sizable portion of no-HS patients (between 42.3 and 58.6% dependent on study), were able to achieve seizure freedom following surgery in majority of the clinical correlation studies conducted with no-HS patients (Davies et al., 1996; de Lanerolle et al., 2003; Blumcke et al., 2007; Thom et al., 2010). This observation possibly indicates that a neuropathological explanation for the epileptogenic focus is present within the resected tissue that is not able to be detected through standard diagnostic practices, making further study of these surgical tissues compelling.

Within mossy fiber sprouting research, two main studies exist which systematically examine the histopathological and clinical correlations of hippocampal mossy fiber sprouting in TLE patients. Proper et al. (2001) conducted the first study in 16 pharmaco-resistant TLE patients. The main finding from this relatively small study was the mossy fiber density in relation to HS Wyler grade, with mossy fiber sprouting into the supragranular layer becoming prominent in moderate to high-grade HS (Wyler 3 and Wyler 4), while mossy fiber density within CA4 decreased in high-grade HS only (Wyler 4) (Proper et al., 2001). This pattern of mossy fiber reorganization likely reflects the progressive loss of mossy fiber target cells in CA3 and CA4, while the mostly preserved granule cells may attract the aberrant axons (Schmeiser et al., 2017). Over a decade later, a 10-fold larger systematic study of mossy fiber sprouting was conducted by Schmeiser et al. (2017) (*n* = 319). This study observed that mossy fiber sprouting pattern did not have any predictive value for post-operative outcome in terms of seizure freedom at years 1–5 post surgery. They were, however, able to make a clinical correlation between mossy fiber sprouting presence, as well as granule cell dispersion and loss of mossy fibers within CA4, and higher age at surgery and increased epilepsy duration prior to surgery (Schmeiser et al., 2017). A correlation was also seen between a high frequency of complex partial seizures and increased mossy fiber sprouting, however this observation did not extend to other seizure types (Schmeiser et al., 2017). Histopathologically, a positive correlation between extent of mossy fiber sprouting and granule cell layer dispersion was also observed (Schmeiser et al., 2017).

## Conclusion

The almost certain co-existence of reactive gliosis with HS has resulted in the complex relationship of these characteristics and TLE to be increasingly investigated and discussed within the literature. Astrocytes have traditionally been thought of as neuron-supporting cells, however, astrocytes are now being recognized to have a much more diverse role within the CNS. Mounting evidence has shown that astrocytes may have a role in HS associated TLE through several cellular pathways including mTOR pathway, gliotransmission, GABA, Aqp4, and GJs. The discussed cellular pathways are not an exhaustive list, but reflect what has been recently pertinent and supported in the literature surrounding astrocyte role in HS associated TLE. Astrocyte role in TLE is especially intriguing in the context of the no-HS subtype of HS associated TLE, as no other histopathological diagnosis can be made aside from reactive gliosis. This lone histological observation in no-HS, along with the tendency of no-HS patients’ condition to improve after surgical resection of the epileptogenic hippocampus points toward a neuropathological explanation for the epileptogenic focus within the resected tissue. Further investigation into HS subtypes and their relationship with associated reactive gliosis is needed to continue to elucidate astrocytes role in HS associated TLE.

## Author Contributions

CT and QZ contributed to the conceptualization and writing and editing the original draft. RA contributed in writing and editing the manuscript. All authors contributed to the article and approved the submitted version.

## Conflict of Interest

The authors declare that the research was conducted in the absence of any commercial or financial relationships that could be construed as a potential conflict of interest.

## Publisher’s Note

All claims expressed in this article are solely those of the authors and do not necessarily represent those of their affiliated organizations, or those of the publisher, the editors and the reviewers. Any product that may be evaluated in this article, or claim that may be made by its manufacturer, is not guaranteed or endorsed by the publisher.
